# Multiple clinical episodes of *Plasmodium falciparum* malaria in a low transmission intensity setting: exposure versus immunity

**DOI:** 10.1186/s12916-015-0354-z

**Published:** 2015-05-13

**Authors:** Josea Rono, Anna Färnert, Linda Murungi, John Ojal, Gathoni Kamuyu, Fatuma Guleid, George Nyangweso, Juliana Wambua, Barnes Kitsao, Ally Olotu, Kevin Marsh, Faith HA Osier

**Affiliations:** KEMRI-Wellcome Trust Research Programme, Centre for Geographical Medicine Research-Coast, Kilifi, Kenya; Infectious Diseases Unit, Department of Medicine, Solna, Karolinska Institutet, Stockholm, Sweden; Centre for Clinical Vaccinology and Tropical Medicine, Churchill Hospital, University of Oxford, Oxford, UK

**Keywords:** Clinical malaria, Immunity, Over-dispersion, Antibodies, Merozoite, *Plasmodium falciparum*

## Abstract

**Background:**

Epidemiological studies indicate that some children experience many more episodes of clinical malaria than their age mates in a given location. Whether this is as a result of the micro-heterogeneity of malaria transmission with some children effectively getting more exposure to infectious mosquitoes than others, or reflects a failure in the acquisition of immunity needs to be elucidated. Here, we investigated the determinants of increased susceptibility to clinical malaria by comparing the intensity of exposure to *Plasmodium falciparum* and the acquisition of immunity in children at the extreme ends of the over-dispersed distribution of the incidence of clinical malaria.

**Methods:**

The study was nested within a larger cohort in an area where the intensity of malaria transmission was low. We identified children who over a five-year period experienced 5 to 16 clinical malaria episodes (children at the tail-end of the over-dispersed distribution, *n* = 35), remained malaria-free (*n* = 12) or had a single episode (*n* = 26). We quantified antibodies against seven *Plasmodium falciparum* merozoite antigens in plasma obtained at six cross-sectional surveys spanning these five years. We analyzed the antibody responses to identify temporal dynamics that associate with disease susceptibility.

**Results:**

Children experiencing multiple episodes of malaria were more likely to be parasite positive by microscopy at cross-sectional surveys (*X*^2^ test for trend 14.72 *P* = 0.001) and had a significantly higher malaria exposure index, than those in the malaria-free or single episode groups (Kruskal-Wallis test *P* = 0.009). In contrast, the five-year temporal dynamics of anti-merozoite antibodies were similar in the three groups. Importantly in all groups, antibody levels were below the threshold concentrations previously observed to be correlated with protective immunity.

**Conclusions:**

We conclude that in the context of a low malaria transmission setting, susceptibility to clinical malaria is not accounted for by anti-merozoite antibodies but appears to be a consequence of increased parasite exposure. We hypothesize that intensive exposure is a prerequisite for protective antibody concentrations, while little to modest exposure may manifest as multiple clinical infections with low levels of antibodies. These findings have implications for interventions that effectively lower malaria transmission intensity.

**Electronic supplementary material:**

The online version of this article (doi:10.1186/s12916-015-0354-z) contains supplementary material, which is available to authorized users.

## Background

Heterogeneity in the risk of *Plasmodium falciparum* malaria in malaria-endemic areas has long been recognized as a common feature of the epidemiology of malaria [1]. Recently, this phenomenon has been described by studies in Senegal [2], Uganda [3] and Kenya [4,5] as well as in large datasets drawn from 90 populations in Africa [6]. In Senegal a subset of children experienced up to twenty malaria episodes in their first two years of life while their age- and location-mates experienced only one episode over the same period [2]. Analysis of the distribution of malaria in a longitudinally monitored population in Kenya revealed that the incidence of malaria was heterogeneous and followed a negative binomial distribution, a phenomenon that was described as over-dispersion [5]. Heterogeneity in infection burden is also evident in other infectious diseases where a small proportion (approximately 20%) of the population is intensely infected and responsible for about 80% of the infectious agent’s transmission, an observation referred to as the ‘20/80’ rule [7].

The factors underlying the heterogeneous epidemiology of malaria are not fully understood. The heterogeneity has been partly attributed to differences in: human genetic [3] and behavioral [8] factors, distance to mosquito breeding grounds [3,9,10], household-related factors [9] and human-mosquito interactions [11]. However, whether children at the tail end of the over-dispersed distribution of malaria differ from children experiencing fewer malaria attacks in their ability to acquire immunity to malaria, as assessed by antibody responses to *P. falciparum* antigens is unknown.

Here, we describe the temporal dynamics of anti-merozoite antibodies in children who were part of the Kenyan cohort described above [5] and differing in their incidence of malaria to determine whether failure to acquire antibodies against these antigens may explain the differences in susceptibility to malaria. We identified, within this cohort and during a five-year follow up period, children who: experienced 5 to 16 episodes of clinical malaria (children at the tail end of the over-dispersed distribution and hereafter referred to as the ‘multiple-episodes’ group), did not experience clinical malaria (‘malaria-free’ group) or had only one episode of clinical malaria (‘single-episode’ group). We then measured antibodies to seven merozoite antigens in these children at six cross-sectional surveys spanning the five-year period and compared the temporal dynamics of anti-merozoite antibodies.

## Methods

### Study population

**Figure 1:**
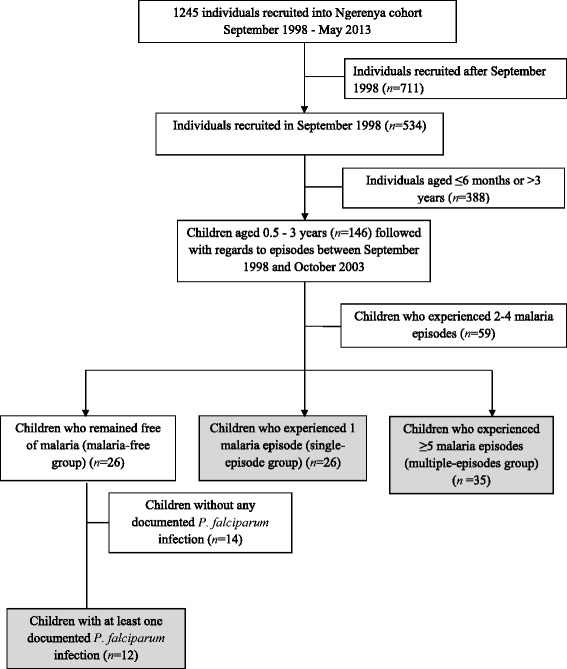
Inclusion of children into the malaria-free, single-episode and multiple-episodes groups. The gray-shaded boxes indicate the number of children included in the three groups investigated in this study.

The study was conducted within a longitudinally monitored population in Ngerenya, located within Kilifi District at the Kenyan coast [5,12]. This population has been monitored from 1998 to date. During this time parasite prevalence declined dramatically such that by 2009 parasite prevalence was zero and has remained so since (Additional file 1: Figure S1). The present report focuses on a subset of children (Figure 1) who were 0.5- to 3-years old in September 1998 (and 5.5- to 8-years old in October 2003) so as to capture the period during which considerable buildup of naturally-acquired anti-merozoite antibodies has been observed in this cohort [13]. During this period there was active weekly surveillance of the cohort and malaria episodes were recorded by active and passive case detection [12]. At the weekly visits children were tested for malaria parasites only if they were symptomatic and treated if parasitemic. In the present analysis, a case of clinical malaria was defined as fever (axillary temperature ≥37.5°C) and any level of parasitemia for children <1-year old and fever accompanied by parasitemia of ≥2,500 parasites/μl of blood for children ≥1-year old [12]. During the same period, six cross-sectional surveys (in September 1998, October 2000, May 2002, October 2002, May 2002 and October 2003) were conducted before the high malaria transmission seasons at which venous blood was collected, and plasma and packed cells stored. At each survey, thick and thin blood smears were prepared and axillary temperature recorded for all participants. Children who were parasitemic at the surveys were not treated for malaria unless they were also symptomatic. Ethical approval for this study was obtained from the KEMRI National Ethics Committee and written informed consent was obtained from the guardians of all children. We compared antibody levels in this cohort (the October 2000 sample) to those in other cohorts we have previously studied [14,15]. Ethical approval for this latter analysis was not required.

### Determination of the malaria exposure index

The malaria exposure index estimates a distance-weighted local prevalence of malaria infection within a kilometer radius around an index child [16]. In essence, an individual’s level of exposure is inferred not from their own history, but from that of the children surrounding them. Children with a high exposure index are more likely to be surrounded by malaria infected children, while those with a low exposure index are not. The malaria exposure index had previously been calculated for the children included in this study [16].

### Detection of *P. falciparum* by microscopy

The detection of *P. falciparum* in whole blood samples collected at the cross-sectional surveys has been described previously [12]. Briefly, thick and thin blood slides were examined by microscopy and parasite densities determined as the number of parasites per 8,000 white blood cells/μL of blood.

### Genotyping of *P. falciparum* infections

Genomic DNA was extracted from packed erythrocytes using QiaAmp Blood Mini kit (Qiagen, Crawley, UK). Genotyping of *P. falciparum msp2* gene was performed as previously described [17]. Briefly, the PCR included an initial amplification of the outer *msp2* domain, followed by nested reactions with fluorescently labeled primers targeting the FC27 and IC-1/3D7allelic types of *msp2*. Fragment sizes were determined by capillary electrophoresis and analyzed using GeneMapper software (Applied Biosystems).

### Recombinant *P. falciparum* merozoite antigens

Five recombinant antigens representing four vaccine candidate antigens were expressed in *Escherichia coli*. The 19-kilodalton fragment of merozoite surface protein (MSP) one-1 (MSP-1_19_) [18], *P. falciparum* reticulocyte-binding homologue 2 (*Pf*Rh2) [19], and two allelic forms of MSP-2: MSP-2_Dd2 (corresponding to the FC27 *msp2* allelic family) and MSP-2_CH150/9 (corresponding to the IC-1 *msp2* allelic family) [20] were expressed as glutathione-S-transferase-fusion proteins. Recombinant MSP-3_3D7 antigen was expressed as a maltose-binding protein-fusion protein [21]. Apical merozoite antigen 1 (AMA-1) from the 3D7 and FVO strains was expressed in *Pichia pastoris* as 6xHis-fusion proteins [22] and generously provided by Dr. Edmond Remarque. Apart from *Pf*Rh2 and MSP-1_19_, all antigens were expressed as full-length proteins.

### Multiplex bead-based antibody assay

Plasma immunoglobulin G (IgG) to the recombinant antigens was measured using a previously described multiplex bead-based assay [15]. Serially-diluted malaria-immune globulin (MIG) [23] was included in each plate as a standard positive control, allowing for the conversion of mean fluorescent intensities to relative antibody concentrations in arbitrary units (AUs) and correction of inter-plate variation. Negative controls, consisting of pooled plasma from adult *P. falciparum* unexposed donors residing in the United Kingdom, were included in each plate to allow for the determination of seropositivity cut-offs. The seropositivity cut-off was determined as the mean fluorescent intensity (MFI) of the negative control plus two standard deviations.

### Data analysis

Data analysis was performed using STATA 11.2. Antibody titers measured in this study were compared to threshold antibody concentrations. These are antibody concentrations against individual antigens measured that appeared to be associated with protection against clinical episodes of malaria in two independent cohort studies [14,15], and were calculated using a standard reference reagent. The threshold antibody concentrations for antibody responses to MSP-1_19,_ MSP-2, MSP-3_3D7 and AMA-1 antigens were 59, 19, 16 and 55 AUs, respectively [14,15]. Antibody titers in children included in this study were also compared to age-matched children in two independent cohorts: the Chonyi cohort in Kenya with *Pf*PR_2–10_ of 44% [14] and a cohort in Rufiji District, Tanzania with *Pf*PR_2–10_ of 49% [15,24]. The P*f*PR_2–10_ is a measure of malaria transmission intensity at a population level [25-28].

Differences in rates of change in antibody titers over the study period were tested using a multilevel mixed-effects linear regression model that accounts for inherent correlations between repeated measurements done on the same subject [29]. In this model, differences in the rates of change of antibody titers in the single-episode and multiple-episodes groups were estimated relative to the malaria-free group and reported as coefficients. The model also took into account the presence or absence of asymptomatic parasitemia at each cross-sectional survey and the number of times an individual was parasitemic during the five year longitudinal follow up.

## Results

**Figure 2:**
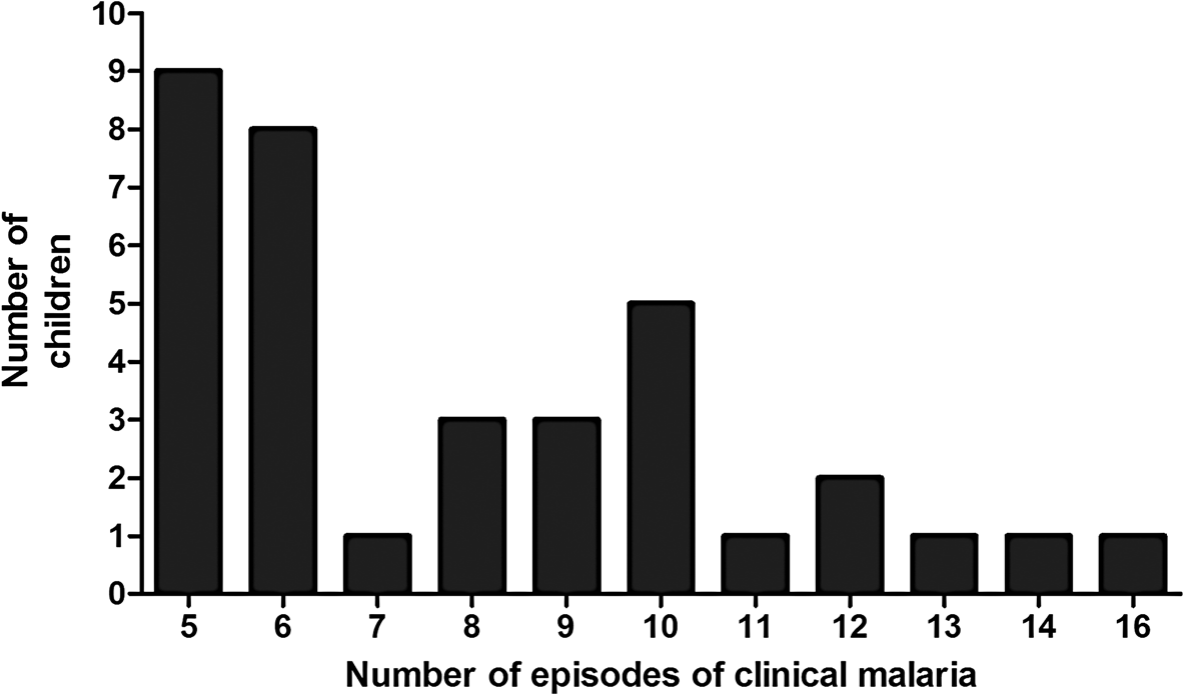
Distribution of clinical malaria episodes per child among children in the multiple-episodes group. The histogram shows the number of children (y axis) within the multiple-episodes group with a given number of clinical malaria episodes (x axis) between September 1998 and October 2003.

In total 1,245 individuals were recruited into the Ngerenya cohort between September 1998 and May 2013. The present study was restricted to the follow up time between September 1998 and October 2003 because this was a five-year period when, compared to other years, *P. falciparum* transmission was highest (Additional file 1: Figure S1). In September 1998 the cohort included 534 individuals (1-month to 82-years old) of which 146 were children 0.5- to 3-years old (Figure 1). Out of the 146 children, 26 had no record of clinical malaria between September 1998 and October 2003; of these 12 had at least one documented asymptomatic *P. falciparum* infection as determined by microscopy (either at cross-sectional surveys or weekly follow up visits) and were classified into the malaria-free group (Figure 1). The remaining 14 were not studied further as exposure to *P. falciparum* parasites could not be definitively ascertained. Twenty six children experienced one episode of malaria in the five-year follow up period and were classified into the single-episode group (Figure 1) while 35 children experienced five to sixteen malaria episodes (Figure 2) and were classified into the multiple-episodes group (Figure 1). The average incidence of malaria was 0.2 and 1.59 (range: 1 to 3.2) episodes/person/year in the single-episode and multiple-episodes groups, respectively. Children who experienced two to four episodes of malaria in the five years of follow up (*n* = 59) were excluded from the present analysis so as to allow for a comparison of antibody responses in children at the extrem**e** ends of the over-dispersed clinical malaria incidence distribution (Figure 1). The age profiles of the study groups were comparable (Table 1).

**Table 1:** **Baseline characteristics**

	**Group of children**		
**Characteristic**	**Malaria-free**	**Single-episode**	**Multiple-episodes**
Number	12	26	35
Age, median age in years (IQR)	1.67 (1.36 to 2.12)	1.52 (1.21 to 2.21)	1.78 (1.38 to 2.41)
Sex, number female (%)	4 /12 (33.33)	8/26 (30.77)	15/35 (42.86)
Hemoglobin AS, number (%)^a,b^	3 (27.27)	4 (17.39)	1 (3.13)
Proportion of cross-sectional surveys at which children were parasite positive by microscopy, proportion (%)	7.5	8	24.26
Malaria exposure index, median (IQR)^c^	0.39 (0.31 to 0.50)	0.54 (0.47 to 0.73)	0.65 (0.52 to 0.76)

^a^Data available for 11, 23 and 32 children in the malaria-free, single-episode and multiple-episodes groups respectively. ^b^There was a non-significant trend towards a larger proportion of children in the malaria-free group compared to single-episode and multiple-episodes groups having the sickle cell trait (Fisher’s exact test *P* = 0.054). ^c^Data available for 10, 21 and 24 children in the malaria-free single-episode and multiple-episodes groups respectively. Age is at the first sampling point in September 1998. IQR, interquartile range.

### Distribution of exposure to *Plasmodium falciparum* parasites in the three study groups

**Figure 3:**
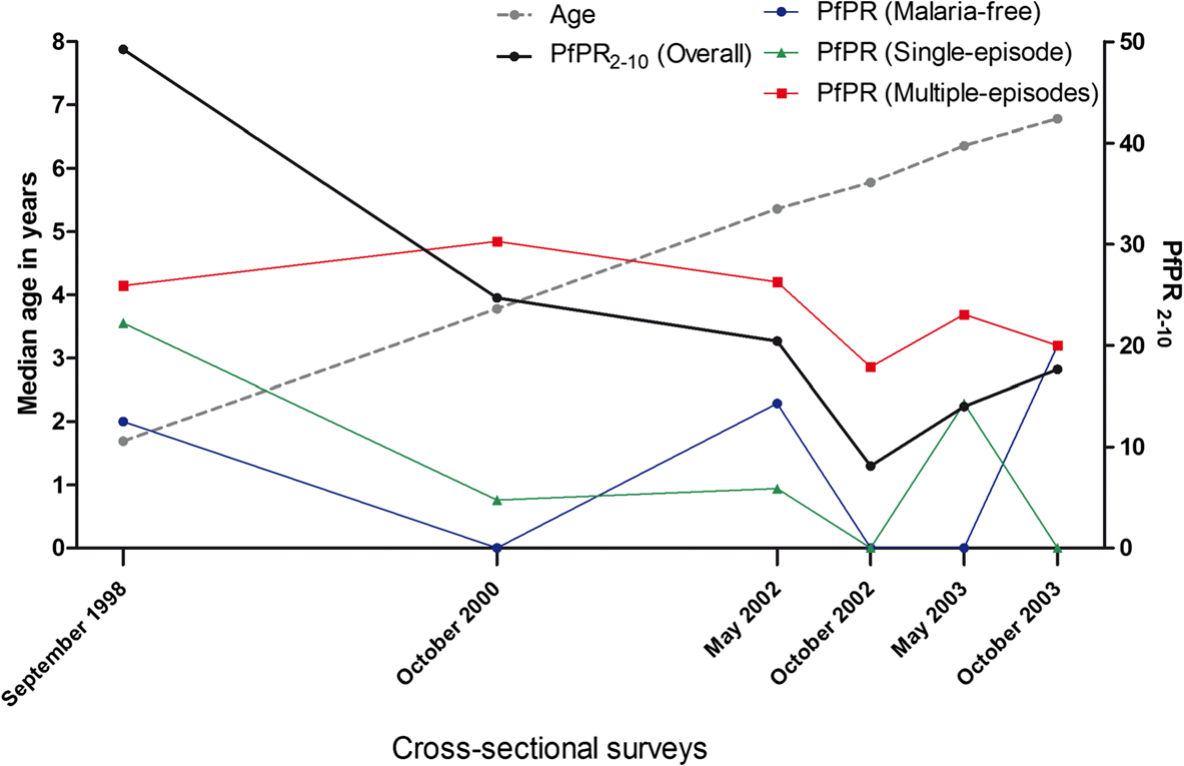
Temporal change in age and parasite prevalence during the study period. The plot shows the median age in years (left y axis) of the children included in this study, the parasite prevalence rates in malaria-free (blue circles), single-episode (green triangles) and multiple-episodes (red squares) groups of children as well as the overall parasite prevalence (black circles) in children 2- to 10-years old (*Pf*PR _2–10_) in the entire Ngerenya cohort at the six cross-sectional surveys.

The proportion of surveys at which children were parasitemic by microscopy was larger in the multiple-episodes group compared to the single-episode and malaria-free groups of children (*X*^2^ test for trend 14.72 *P* = 0.001, Table 1). Children in the multiple-episodes group had higher malaria exposure, as measured by the malaria exposure index (a distance-weighted local prevalence of malaria) [16], compared to children in the single-episode and malaria-free groups (Kruskal-Wallis test *P* = 0.009, Table 1). The overall *Pf*PR_2–10_ in the Ngerenya cohort decreased over the study period with the decrease being more evident in the malaria-free and single-episode groups of children compared to the multiple-episodes group (Figure 3). *P. falciparum* parasites were detected by PCR in 26.7%, 19.5% and 24.4% of the children at the September 1998, October 2000 and May 2002 cross-sectional surveys, respectively. The number of *msp2* genotypes, that is, clones in PCR positive samples at the cross-sectional surveys, ranged from one to four, and was higher in the multiple-episodes group than in the single-episode and malaria-free groups (Additional file 1: Figure S2).

### Antibody profiles of individual children over time

**Figure 4:**
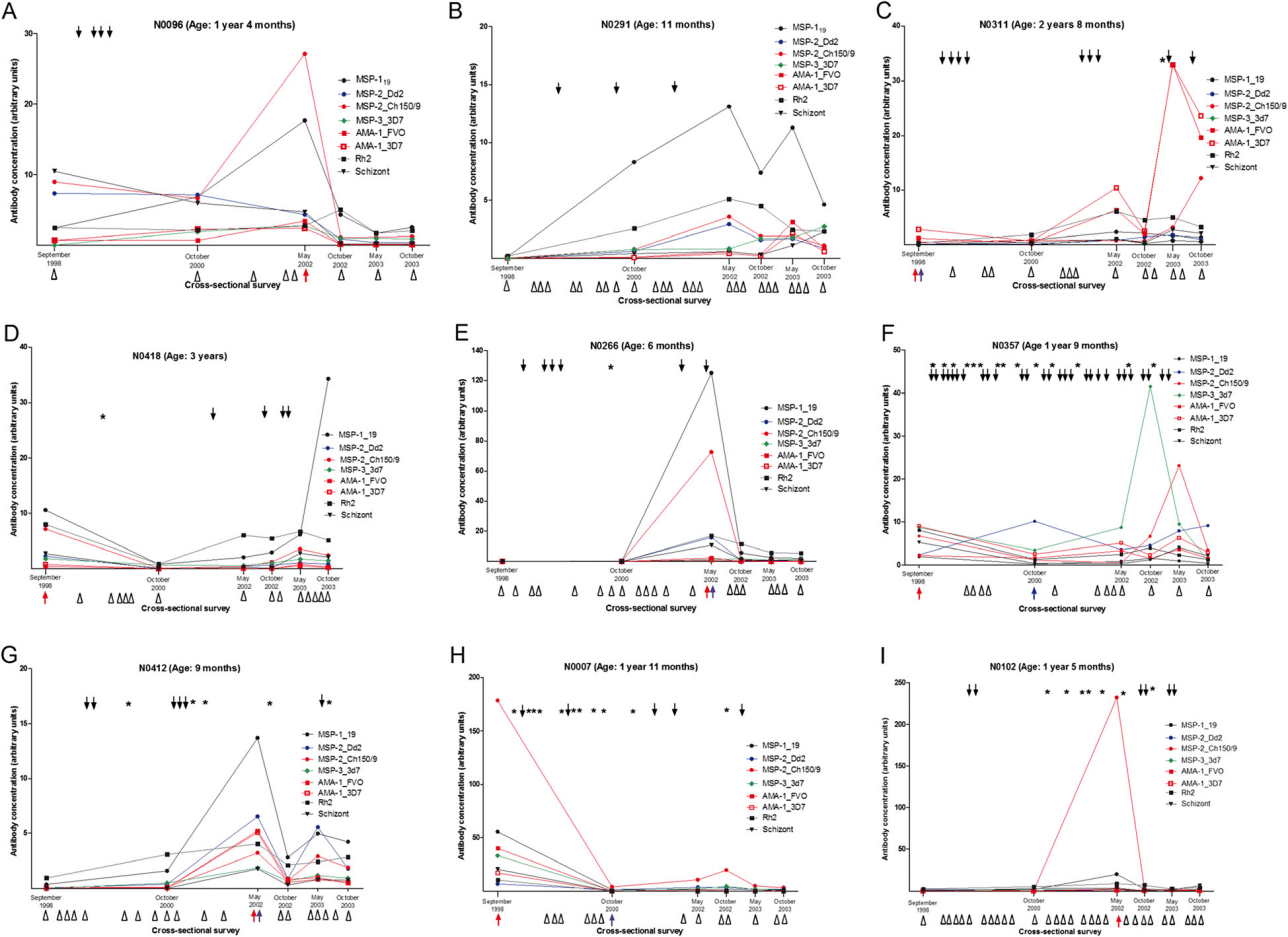
Antibody and *P. falciparum* infection profiles of individual children. The plots show the levels of IgG antibodies (y axis) to a panel of merozoite antigens at each of the six cross-sectional surveys (x axis) conducted between 1998 and 2003. The black solid arrows indicate the time during follow up when an individual child was parasitemic by microscopy. Asterisks indicate the time during follow up when a child had an episode of clinical malaria. The open triangles along the x axis indicate either the cross-sectional surveys when a child was aparasitemic or the weekly follow up visits when a child was symptomatic but found to be aparasitemic by microscopy. Red and blue arrows along the x-axis indicate the cross-sectional surveys at which a child was infected with *P. falciparum* clones of the IC-1 or FC *msp2* types, respectively. Panel **A**-**B**, **C**–**E** and **F**–**I** show the profiles of children belonging to the malaria-free, single-episode and multiple-episodes groups, respectively. Ages are reported as at baseline, that is, in September 1998. IgG, immunoglobulin G.

Diverse longitudinal antibody profiles were observed in individual children across the three groups (Figure 4). Some children maintained relatively low antibody titers to all the antigens and only had relatively high titers at those cross-sectional surveys at which they were parasitemic (Figure 4A, E, G, H and I). In children with these profiles, there was an indication of allele-specific boosting of anti-MSP-2 antibody responses; infections with clones of IC-1 or FC *msp2* types were associated with high antibody titers to the corresponding MSP-2 type antigen (Figure 4A, F and H). For instance, the IC-1 infection in May 2002 in child N0102 (Figure 4I) coincides with high titers to MSP-2_Ch150/9 but not to MSP-2_Dd2. Some children, however, had profiles characterized by relatively high antibody titers to some antigens at surveys at which they were aparasitemic (Figure 4B). Remarkably, some children maintained low antibody titers despite having a high intensity of asymptomatic infections and episodes of malaria (Figure 4F and H).

### Comparison of antibody titers at individual cross-sectional surveys in the three study groups

**Figure 5:**
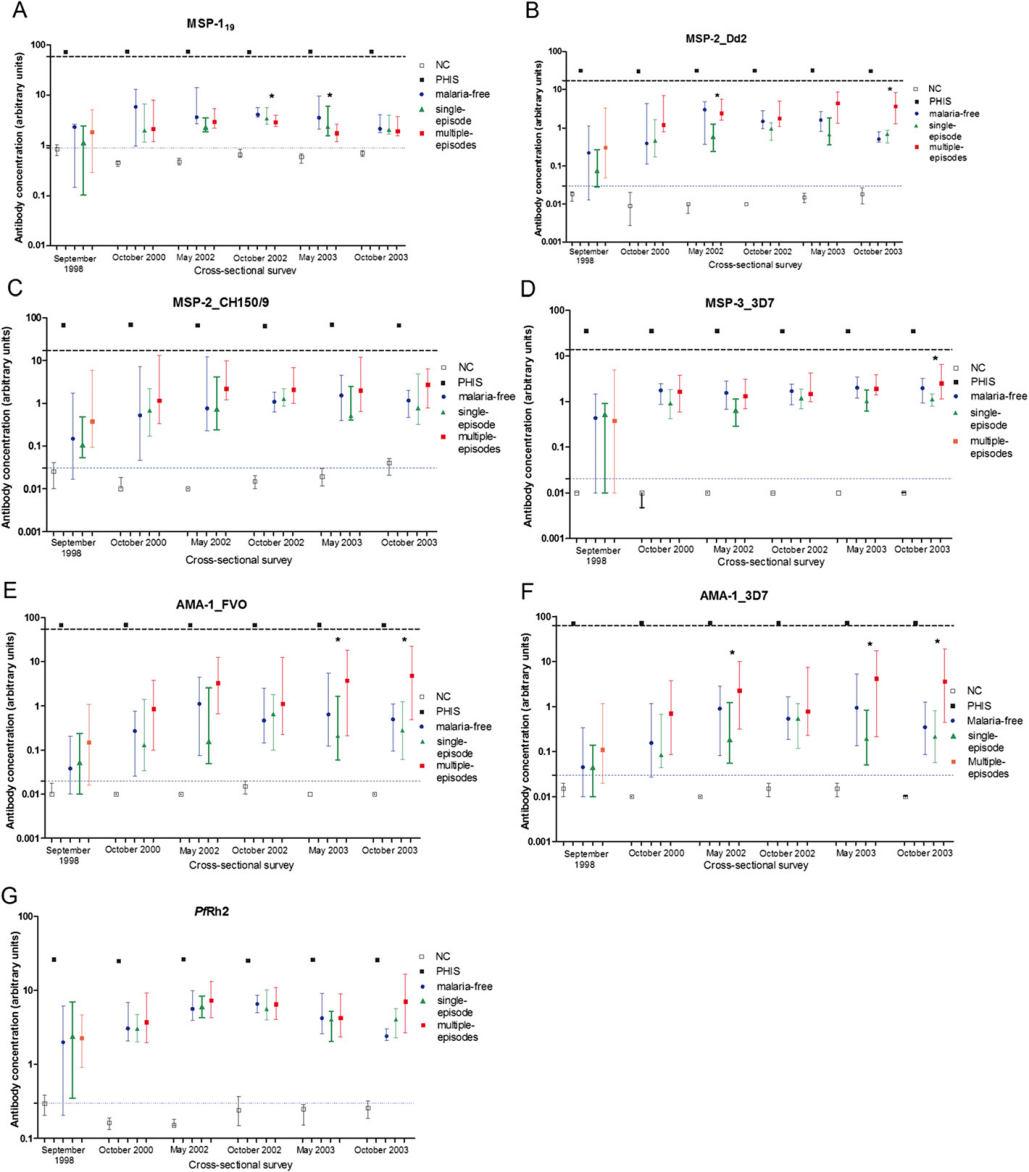
Distribution of antibody titers to individual merozoite antigens among the three groups of children. The panels show the distribution of antibody titers (median and interquartile range) in the malaria-free (blue circles), single-episode (green triangles) and multiple-episodes (red squares) groups of children at six cross-sectional surveys for the respective antigens: **A)** MSP-1_19_, **B)** MSP-2_Dd2, **C)** MSP-2_CH150/9, **D)** MSP-3_3D7, **E)** AMA-1_FVO, **F)** AMA-1_3D7 and **G)** *Pf*Rh2. ‘NC’ refers to antibody titers in sera from *P. falciparum*-naïve adults (used here as negative controls). ‘PHIS’ refers to antibody titers in a pool of hyperimmune sera (used here as a positive control). The black bold dotted line shows the ‘threshold’ antibody concentrations to respective antigens that were calculated as described in the Results section. The thin dotted blue line shows the ‘seropositivity cut off’ based on the mean plus two standard deviations of the antibody titer obtained with the negative control sera.

Comparisons of antibody titers to each of the seven antigens in the three study groups are shown in Figure 5A-5G. Generally, the multiple-episodes group had lower titers to MSP-1_19_ (Figure 5A) but higher titers to MSP-2_Dd2 (Figure 5B), MSP-3_3D7 (Figure 5D), and both AMA-1 alleles (Figure 5E and F) compared to the single-episode and malaria-free groups. To better understand the antibody data, we compared antibody titers to MSP-1_19_, MSP-2 and AMA-1 in the three study groups to titers in age-matched children in the Chonyi [14] and Tanzania [15] cohorts. Antibody titers in children included in this study were generally lower than in age-matched children in the Chonyi and Tanzania cohorts (Additional file 1: Figure S3) and were also below threshold concentrations that appear to be necessary for protection against clinical episodes of malaria [14,15]. Moreover, antibody titers obtained with a pool of hyperimmune sera (PHIS) were well above these threshold concentrations for all the antigens tested (Figure 5).

### Comparisons of the temporal changes in antibody response in the three study groups

At the start of this study (September 1998), antibody titers to all antigens were comparable among the three study groups. Univariate analysis showed evidence for a significantly higher rate of increase in antibody titers to AMA-1_FVO and AMA_3D7 over the five-year period in the multiple-episodes compared to the single-episode group of children (Table 2). After adjusting for both sickle cell trait and asymptomatic parasitemia, the difference in the rate of increase in antibody titers was only evident for AMA-1_FVO (coefficient −6.32, 95% confidence interval (CI) -12.64 to −0.01, Table 2). There were no significant differences in rates of change of antibody titers to other merozoite antigens among the three study groups (Table 2).

**Table 2:** **Temporal change in antibody titers among the three groups of children**

**Antigen**	**Group of children**	**Coefficient (95% CI)**			
		**Unadjusted**	**Adjusted** ^**a**^	**Adjusted** ^**b**^	**Adjusted** ^**c**^
MSP-1_19_	Malaria-free	Reference group	-	-	-
	Single-episode	−2.00 (−11.27 – 7.27)	−2.68 (−12.03 – 6.74)	−1.93 (−10.32 – 6.46)	−2.58 (−11.10 – 5.93)
	Multiple-episodes	−2.54 (−11.37 – 6.28)	−2.54 (−12.91 – 5.59)	−3.82 (−11.85 – 4.20)	−4.93 (−13.34 – 3.49)
	Single-episode versus Multiple-episodes	0.54 (−5.88 – 6.96)	0.99 (−5.52 – 7.50)	1.89 (−4.02 – 7.80)	2.35 (−4.02 – 7.80)
MSP-2_Dd2	Malaria-free	Reference group	-	-	-
	Single-episode	0.43 (−5.01 - 5.86)	0.29 (−5.27 - 5.864)	0.24 (−4.28 - 5.66)	0.19 (−5.17 - 5.55)
	Multiple-episodes	1.90 (−3.01 – 7.35)	1.90 (−3.55 – 7.36)	1.33 (−3.69 – 6.36)	0.94 (−4.34 – 6.23)
	Single-episode versus Multiple-episodes	−1.74 (−5.53– 2.06)	−1.62 (−5.50– 2.26)	−0.93 (−4.65– 2.79)	−0.75 (−4.55– 3.05)
MSP-2_CH150/9	Malaria-free	Reference group			
	Single-episode	0.24 (−9.37 - 9.86)	0.35 (−10.21 - 9.50)	0.41 (−8.77 - 9.59)	0.34 (−9.72 - 9.05)
	Multiple-episodes	6.75 (−2.39 – 15.89)	5.83 (−3.87– 15.53)	3.87 (−4.91– 12.66)	2.68 (−6.62– 11.99)
	Single-episode versus Multiple-episodes	−6.51 (−13.00– 0.02)	−6.18 (−12.79– 0.43)	−3.46 (−12.79– 0.43)	−3.02 (−9.47– 3.42)
MSP-3_3D7	Malaria-free	Reference group	-	-	-
	Single-episode	−2.42 (−7.17 - 2.31)	−2.23 (−7.10- 2.63)	−2.37 (−7.04- 2.30)	−2.37 (−7.03- 2.55)
	Multiple-episodes	−0.50 (−5.02 – 4.02)	−0.20 (−4.98 – 4.57)	−1.20 (−5.67 – 3.26)	−0.99 (−5.72 – 3.72)
	Single-episode versus Multiple-episodes	−1.93 (−5.24 – 1.38)	−2.03 (−5.43 – 1.37)	−1.17 (−4.48 – 2.15)	−1.24 (−4.64 – 2.17)
AMA-1_FVO	Malaria-free	Reference group	-	-	-
	Single-episode	−3.71 (−12.55 - 5.14)	−4.32 (−13.32 – 4.68)	−3.64 (−12.48 – 5.20)	−3.76 (−13.24 – 4.72)
	Multiple-episodes	3.87 (−5.54 – 12.30)	2.87 (−5.97 – 11.71)	3.10 (−5.35 – 11.56)	3.10 (−6.82– 10.94)
	Single-episode versus Multiple-episodes	−7.59 (−13.71 – -1.47)*	−7.19 (−13.40 – -0.98)*	−6.75 (−12.97 – -0.52)*	−6.32 (−12.64 – -0.01)*
AMA-1_3D7	Malaria-free	Reference group			
	Single-episode	−2.83 (−10.37 - 4.71)	−3.43 (−11.09 - 4.23)	−2.79 (−10.30 - 4.72)	−3.37 (−10.99 - 4.25)
	Multiple-episodes	3.17 (−4.02 – 10.35)	2.17 (−5.36 – 9.70)	2.83 (−4.34 – 10.01)	1.85 (−5.68 – 9.38)
	Single-episode versus Multiple-episodes	−5.99 (−11.22 – 0.77)*	−5.60 (−10.90 – -0.31)*	−5.63 (−10.92 – -0.33)*	−5.22 (−10.60 – 1.52)
*Pf*Rh2	Malaria-free	Reference group	**-**	**-**	**-**
	Single-episode	−5.50 (−13.21 - 2.27)	−5.79 (−13.71 - 2.12)	−5.42 (−13.22 - 2.38)	−5.77 (−13.74 - 2.19)
	Multiple-episodes	−1.72 (−9.09 – 5.64)	−2.25 (−10.03 – 5.54)	−1.99 (−9.44 – 5.48)	−2.57 (−10.46 – 5.32)
	Single-episode versus Multiple-episodes	−3.74 (−9.03 – 1.54)	−3.55 (−8.92 – 1.83)	−3.44 (−8.86 – 1.99)	−3.21 (−8.73 – 2.32)

^a^Adjusted for sickle cell trait (hemoglobin AS). ^b^Adjusted for whether a child was parasitemic or aparasitemic by microscopy at each of the six cross-sectional surveys. ^c^Adjusted for sickle cell trait and whether a child was parasitemic or aparasitemic by microscopy at each of the six cross-sectional surveys. The coefficients indicate differences in the rates of change of antibody titers comparing the single- and multiple-episodes groups to the malaria-free group. Antibodies to AMA1_FVO increased significantly over five years when comparing the multiple- to the single-episode group. For all other antigens, the rates of change in antibody titers were not significant during this period. CI, confidence interval. *P < 0.05.

## Discussion

This is, to our knowledge, the first attempt to investigate the underlying immunological determinants of the over-dispersion of clinical episodes of malaria in a low transmission intensity setting. Our study showed that over a five-year period, children who were susceptible to multiple clinical attacks of malaria had higher indices of exposure to infectious mosquitoes than those with single or no episodes. In contrast, the temporal dynamics of antibody responses to *Plasmodium falciparum* merozoite antigens were generally similar in all groups of children. Of note, antibody levels in all groups of children were lower than those previously shown to correlate with protection against clinical malaria. These data suggest that the differences in susceptibility to clinical malaria among children in this context are attributable to differences in exposure to infectious mosquitoes rather than to a failure to acquire immunity, as reflected by anti-merozoite antibodies.

The malaria exposure index [16] estimates a distance-weighted local prevalence of malaria infection within a kilometer radius around an index child. By this measure, children in the multiple-episodes group had more exposure to the parasite compared to children in the malaria-free and single-episode groups. Coupled with the fact that the children in the multiple episode group were also more likely to be parasite positive (asymptomatically parasitemic) and with more clones (parasites of different *msp2* genotypes) at the cross-sectional surveys is a strong indication that children in the multiple episodes groups are indeed more exposed to the parasite. In the absence of clear differences in the acquisition of immunity (as estimated by anti-merozoite antibodies), our data suggest that the differences in disease susceptibility were driven by the observed differences in the intensity of exposure to the parasite.

Malaria transmission intensity was low in the study area. *P. falciparum* exposure (as estimated by *Pf*PR) in the three groups of children studied here did not exceed 30% at any of the cross-sectional surveys over five years. This is relatively low compared to the 40% cutoff above which a population is considered to be under high malaria transmission [25], and is supported by our finding that antibody titers in these children were lower than those of age-matched children in separate cohorts under higher malaria transmission intensity. Furthermore, the incidence rate of malaria in the multiple-episodes group (1.59 episodes/person/year) is less than a third of what has been reported from high malaria transmission areas (5.3 episodes/person/year) [30]. The low intensity of malaria transmission probably accounts for the fact that although children in the multiple-episodes group had higher indices of parasite exposure, their antibody levels were still lower than those known to correlate with protection against clinical episodes of malaria [14,15]. It may also explain why no significant differences in antibody levels were observed between the multiple episodes group and the malaria-free or single-episode groups.

Overall, with the exception of AMA-1-FVO, the rate of change of antibody titers over time did not differ significantly between the three groups. The absence of any significant differences in the temporal change in antibody titers between the groups could be attributable to the young age of the study subjects, small sample size or the antigens studied. It is possible that the young age of the study subjects precludes any potential differences in rates of buildup of antibodies among the study groups. This is plausible considering that, given uniform parasite exposure, the buildup of antibody titers is slower in children than in adults [31], implying that young age is inherently associated with slow acquisition of antibody responses. The present study was limited to a panel of merozoite antigens to which antibody titers have been shown to correlate with protective immunity [32]. The selection of these antigens allowed for the use of antibody titers as proxies of naturally acquired immunity.

Generally, we observed allele-specific boosting of anti-MSP-2 antibodies, which has also been reported in relation to MSP-2 [33] and AMA-1 [34] and suggests that the transient peaks in antibody titers are generated by the differentiation of naive B-cells into short-lived plasma cells (SLPCs) driven by concurrent infection rather than by long-lived plasma cells (LLPCs) generated from previous infections. Indeed, acute malaria infections in children lead to expansion of anti-merozoite antibodies and memory B-cell (MBC) pools which decline in the absence of parasites [35]. The dependence of antibody production in children on SLPCs may explain why antibody titers in the multiple episodes group were not higher than what we observed given the higher frequency of *P. falciparum* infections. Considering that differentiation of MBCs into SLPCs peaks six to eight days after re-exposure to antigen [36], antibody generation following acute infection in children may not be fast enough to prevent the rapid increase in parasitemia and thus clinical malaria which occurs approximately three days after blood-stage infection [37]. The incidence of malaria in children may thus be a reflection of the intensity of parasite exposure in individuals whose LLPCs are insufficiently developed to sustain antibodies at high enough concentrations to confer protection against malaria. However, given that the relative contributions of each *msp2* genotype to the infection could not be determined using capillary sequencing as employed here, interpretation on disease causation and/or immunity should be cautious.

The observed difference in the rates of change of antibody titers to AMA-1-FVO, but not to the other antigens in the three study groups, may be a reflection of antigen-specific differences in human immune responses to different antigens. Antibody longevity [38] and affinity [39] are known to vary between individual merozoite antigens. Nonetheless, the observed difference in the rates of change of antibody titers to AMA-1-FVO raises the question as to whether antibodies to AMA-1 are a good correlate of exposure [40] or of protective immunity [32].

We observed that children in the multiple-episodes group had more genetically-diverse infections compared to children in the single-episode and malaria-free groups. This finding is consistent with previous observations that genetically-diverse infections in children are associated with an increased risk of malaria [15,41,42]. We have previously shown that genetically-diverse infections are more often present in young children who develop severe non-cerebral malaria compared to age- and location-matched children [43]. Taken together with the antibody data presented here, these findings suggest that the genetic diversity of asymptomatic infections in young children is a marker of the intensity of exposure to the parasite at a time when anti-merozoite antibodies have not attained concentrations required for protection against malaria.

In our study, other than at cross-sectional surveys, children were only tested for malaria parasites when they were symptomatic. Thus, it is possible that some asymptomatic infections were undetected, but that would apply equally to all groups and thus is unlikely to introduce bias. Another limitation of the study was the availability of antibody measurements only at the cross-sectional surveys and not when children were symptomatic. Thus, we could not compare antibody levels at the point of symptomatic infections in the multiple- and single-episode groups. We were also unable to account for the possible effects of HIV, malnutrition and prenatal *P. falciparum* exposure on antibody titers in the children studied here [44].

## Conclusions

In summary, our data shows that in the context of a low malaria transmission setting, multiple episodes of clinical malaria are more likely the consequence of increased exposure rather than failure to acquire immunity. We hypothesize that intensive exposure induces antibodies at protective concentrations, while little to modest exposure may manifest as multiple clinical infections with low levels of antibodies. Future studies on the determinants of increased susceptibility to clinical malaria in areas with high malaria transmission intensity will complement the data presented here and help to define the tipping point in malaria transmission intensity where exposure translates into protective immunity as opposed to increased susceptibility to disease. Our data have implications for interventions including vaccines that effectively lower but do not completely abolish malaria transmission intensity. These interventions may reduce malaria transmission intensity below the threshold necessary to induce protective immunity and thus drive increased susceptibility to clinical malaria.

## Additional file

Additional file 1:
**Supplementary figures.** Demonstrate 1) the temporal variation in cohort size and parasite prevalence, 2) the distribution of the number of *P. falciparum msp2* genotypes in the three groups of children and 3) the distribution of antibody titres in age-matched children in the Ngerenya, Chonyi and Tanzania cohorts.

## Abbreviations

AMA-1: apical membrane antigen 1
AUs: arbitrary units
LLPCs: long-lived plasma cells
MBCs: memory B-cells
MFI: mean fluorescent intensity
MIG: malaria immune globulin
MSP: Merozoite surface protein
PHIS: pool of hyperimmune sera
SLPCs: short-lived plasma cells
