# Supplementary material for: Multiple clinical episodes of Plasmodium falciparum malaria in a low transmission intensity setting: exposure versus immunity
Source: BMC Med. 2015 May 13;13:114. doi: 10.1186/s12916-015-0354-z (PMC4445794; doi:10.1186/s12916-015-0354-z)
Supplement: Additional file 1: — Supplementary figures. Demonstrate 1) the temporal variation in cohort size and parasite prevalence, 2) the distribution of the number of P. falciparum msp2 genotypes in the three groups of children and 3) the distribution of antibody titres in age-matched children in the Ngerenya, Chonyi and Tanzania cohorts. [file 12916_2015_354_MOESM1_ESM.doc]

**Supplementary Figure 1. Temporal variation in cohort size and parasite prevalence.**

The plot shows the parasite prevalence in children aged 2-10 years (*Pf*PR_2-10_) (left y axis) and the total number of individuals present (right y axis) at each cross-sectional survey conducted in the Ngerenya cohort between September 1998 and May 2011.

| **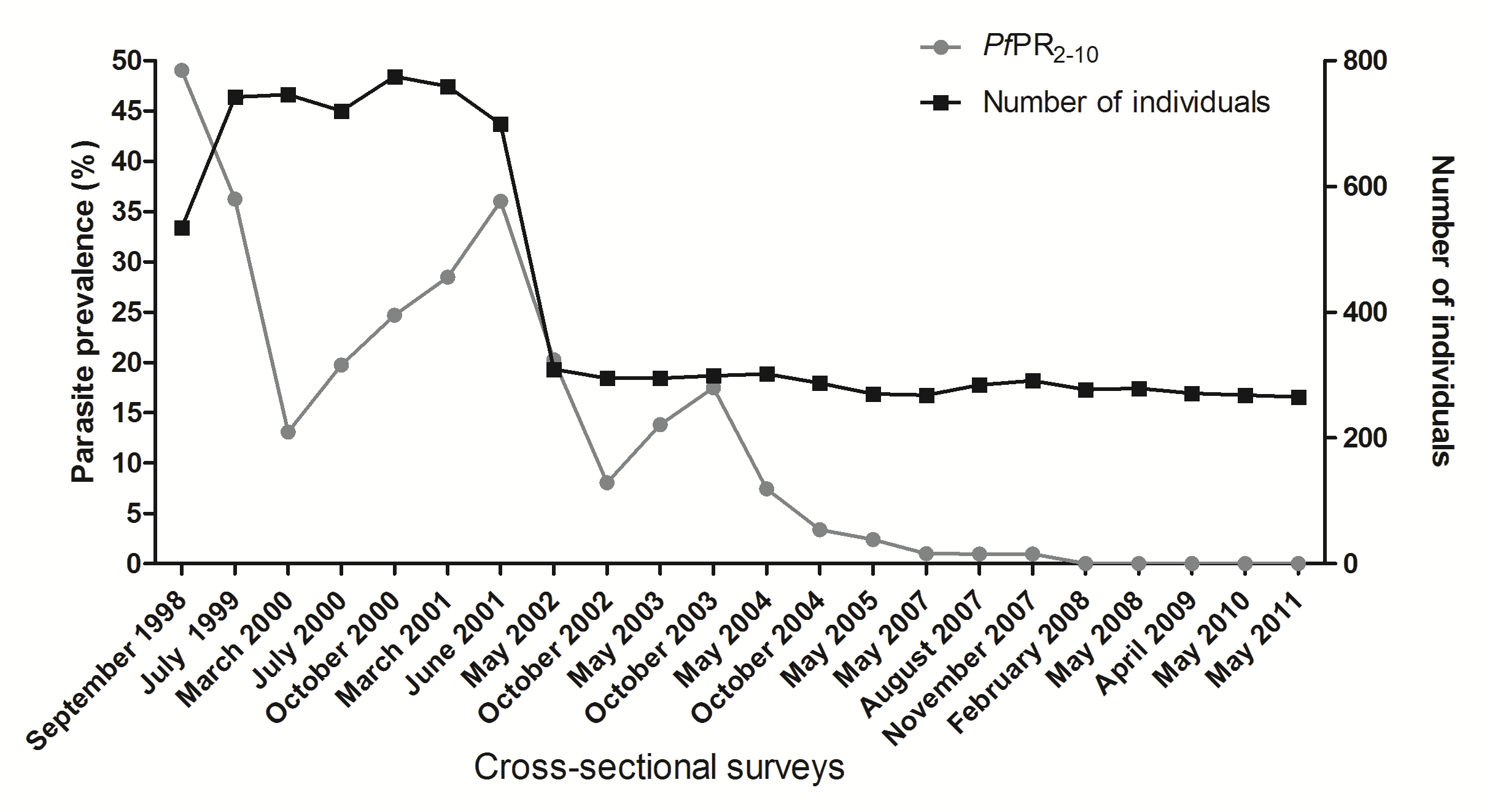** |
| --- |

**Supplementary Figure 2. Distribution of the number of *P. falciparum* *msp2* genotypes in the three groups of children.**

The bar graphs show the number of children within each group with different number of *msp2* genotypes (i.e. clones) at the respective cross-sectional surveys. Data is shown for 45 (6, 17 and 22 in the malaria-free, single-episode and multiple-episodes groups respectively), 41 (6, 13 and 22 in the malaria-free, single-episode and multiple-episodes groups respectively) and 45 (5, 16 and 24 in the malaria-free, single-episode and multiple-episodes groups respectively) children at the September 1998, October 2000 and May 2002 cross-sectional surveys respectively for whom samples for *msp2* typing were available.


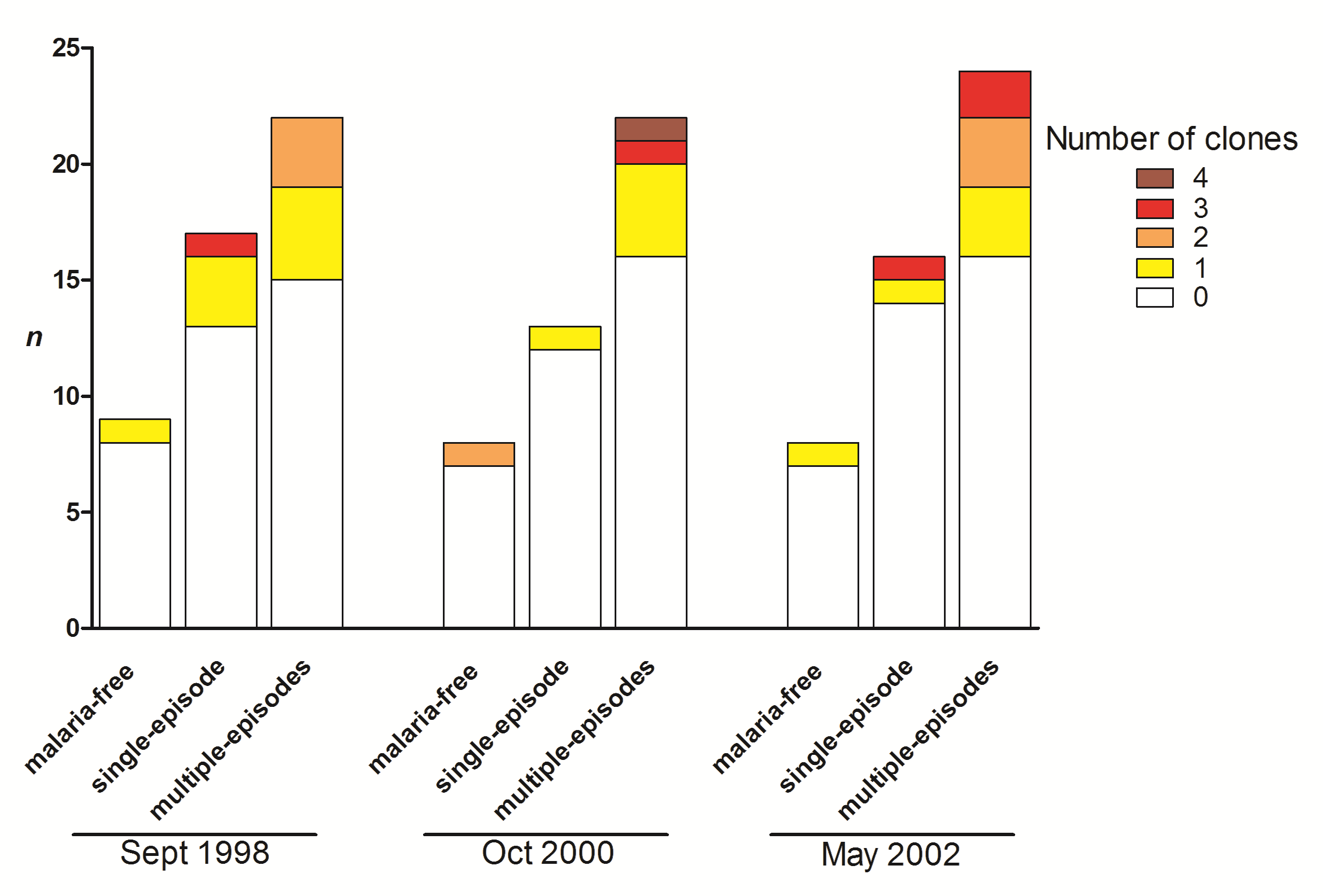


**Supplementary Figure 3. Distribution of antibody titres in age-matched children in the Ngerenya, Chonyi and Tanzania cohorts.**

The panels show the distribution of antibody titres in age-matched children in Ngerenya (blue circles), Chonyi (grey circles) and Tanzania (black circles) cohorts to; A) MSP-1_19_, B) MSP-2_Dd2 C) MSP-3_3D7 and D) AMA-1_FVO. Black lines indicate the median antibody concentrations. Data from the Chonyi cohort are based on sera samples collected in October 2000 when parasite prevalence in children aged 2-10 years (*Pf*PR_2-10_) was 44%. Data from the Tanzania cohort are based on sera samples collected in March-April 1999 when *Pf*PR_2-10_ was 49%. Ngerenya data are based on samples collected in October 2000 when P*f*PR_2-10_ was 25%.

| **A**  **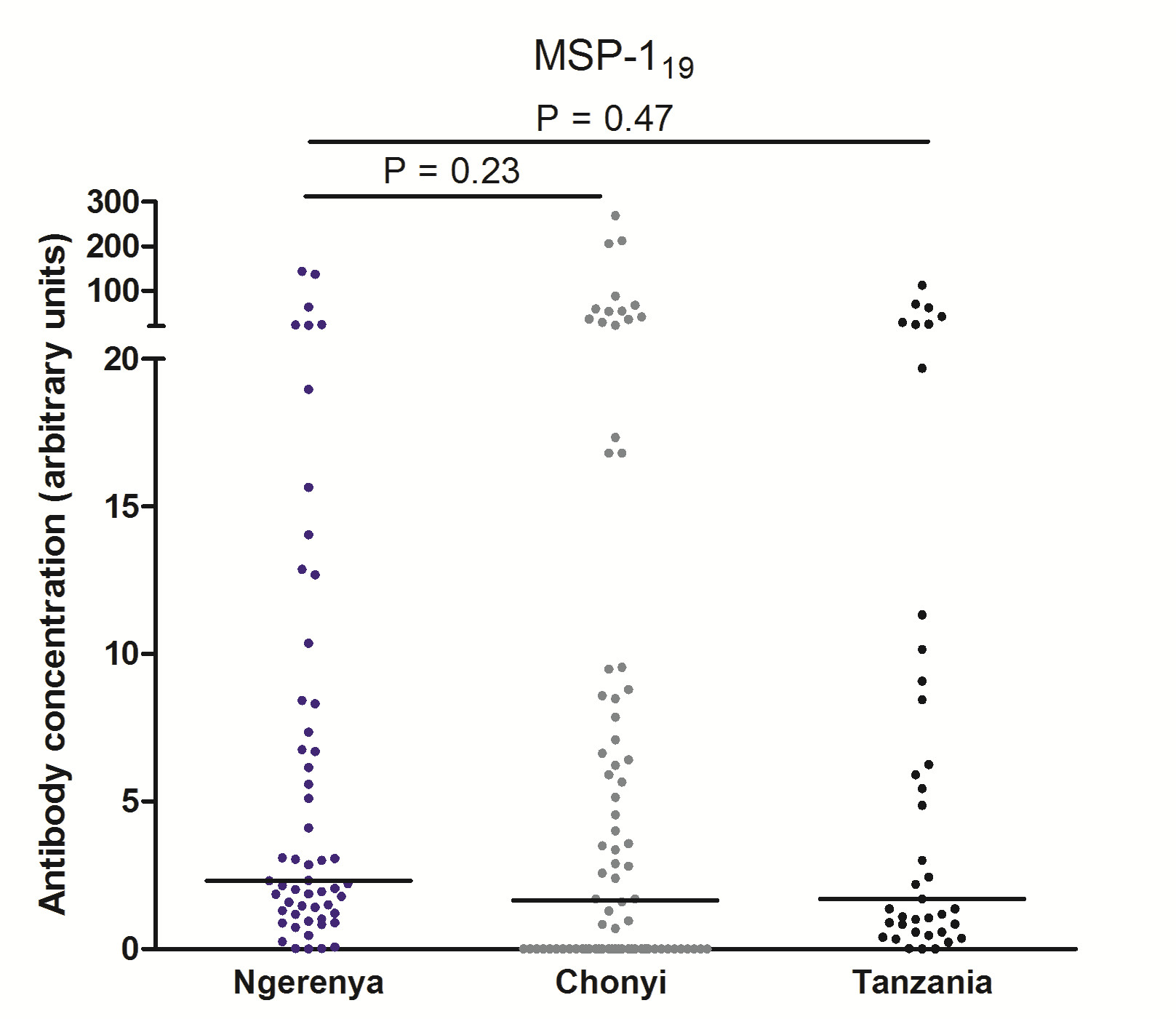** | **B**  **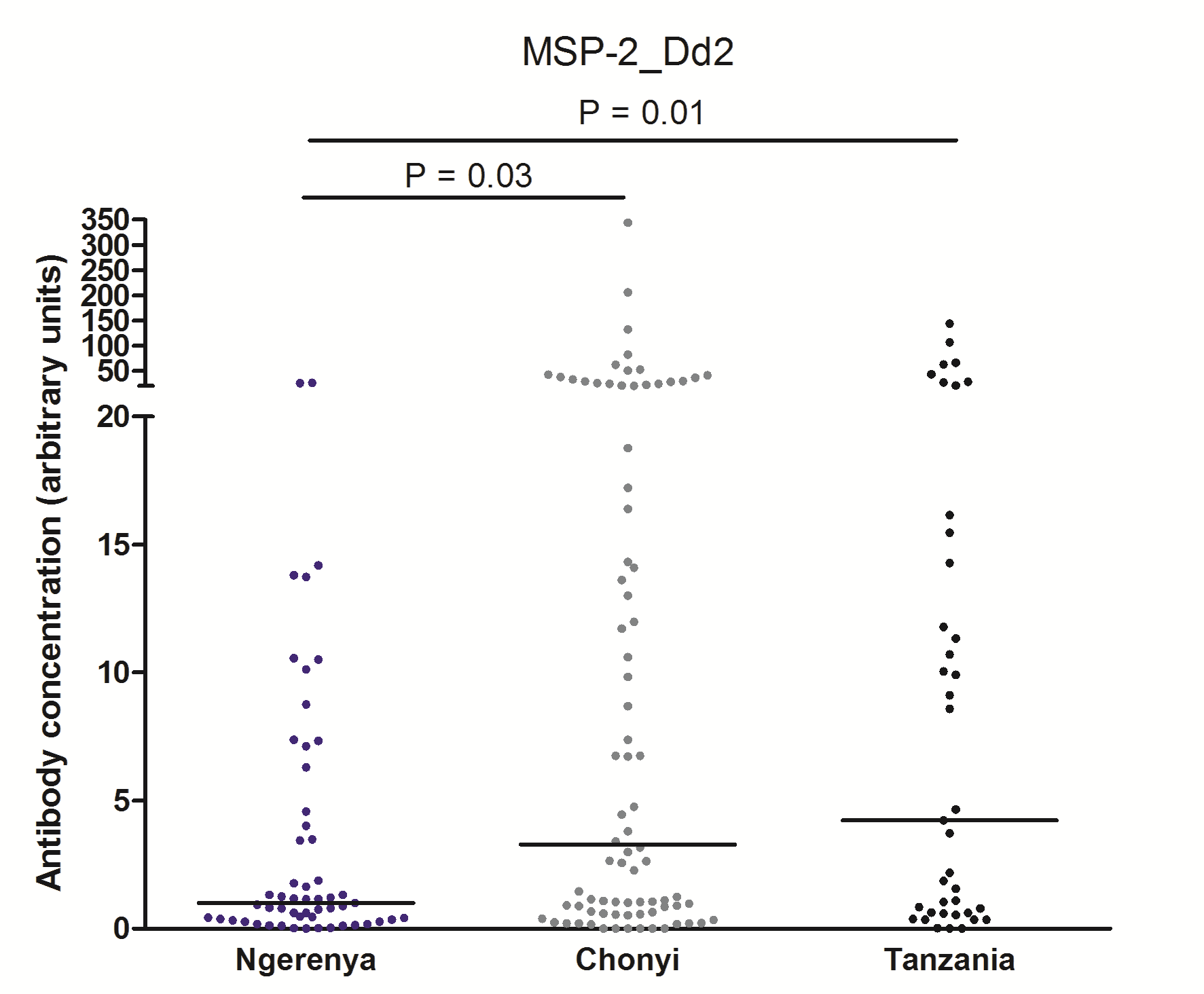** |
| --- | --- |
| **C**  **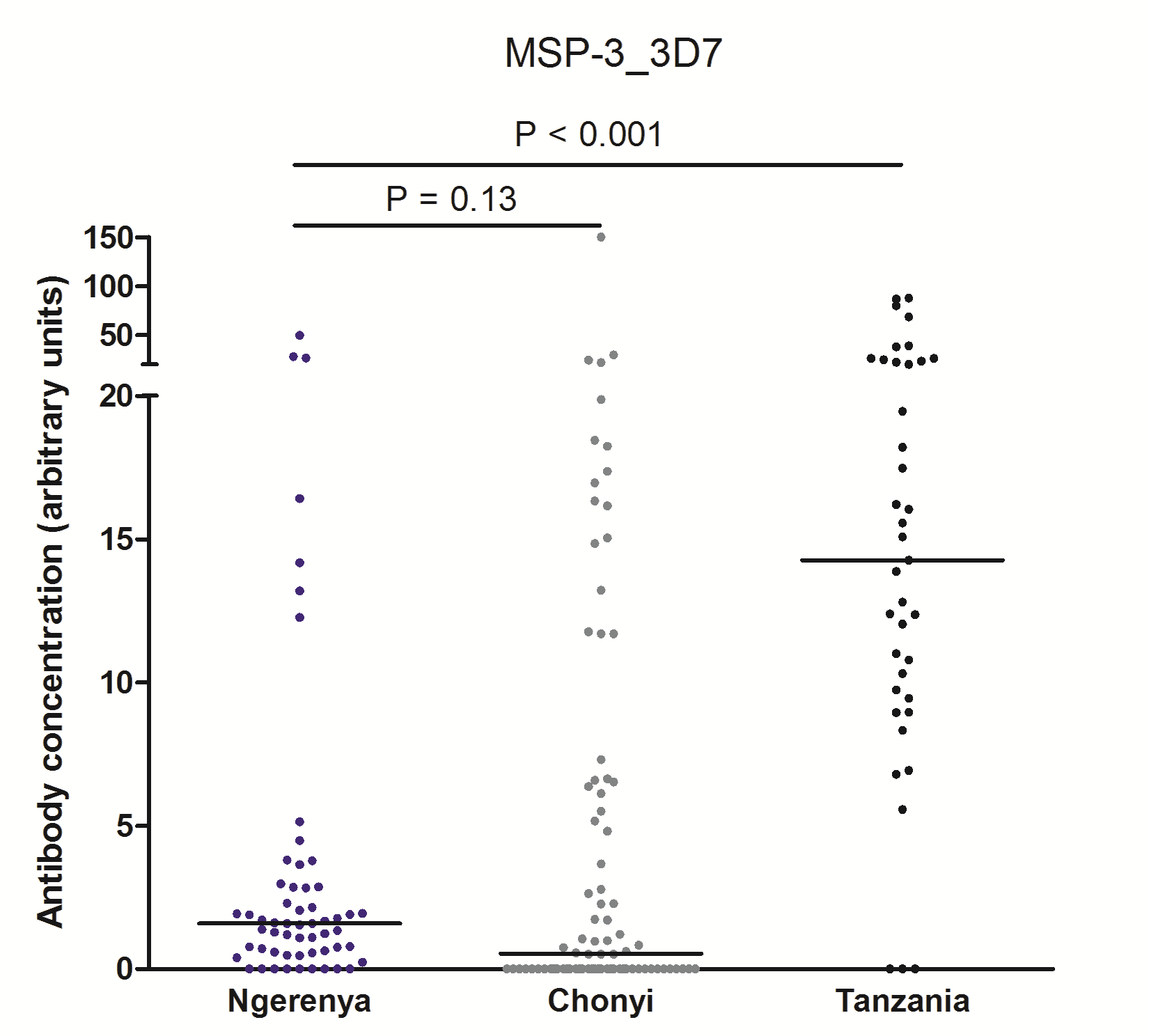** | **D**  **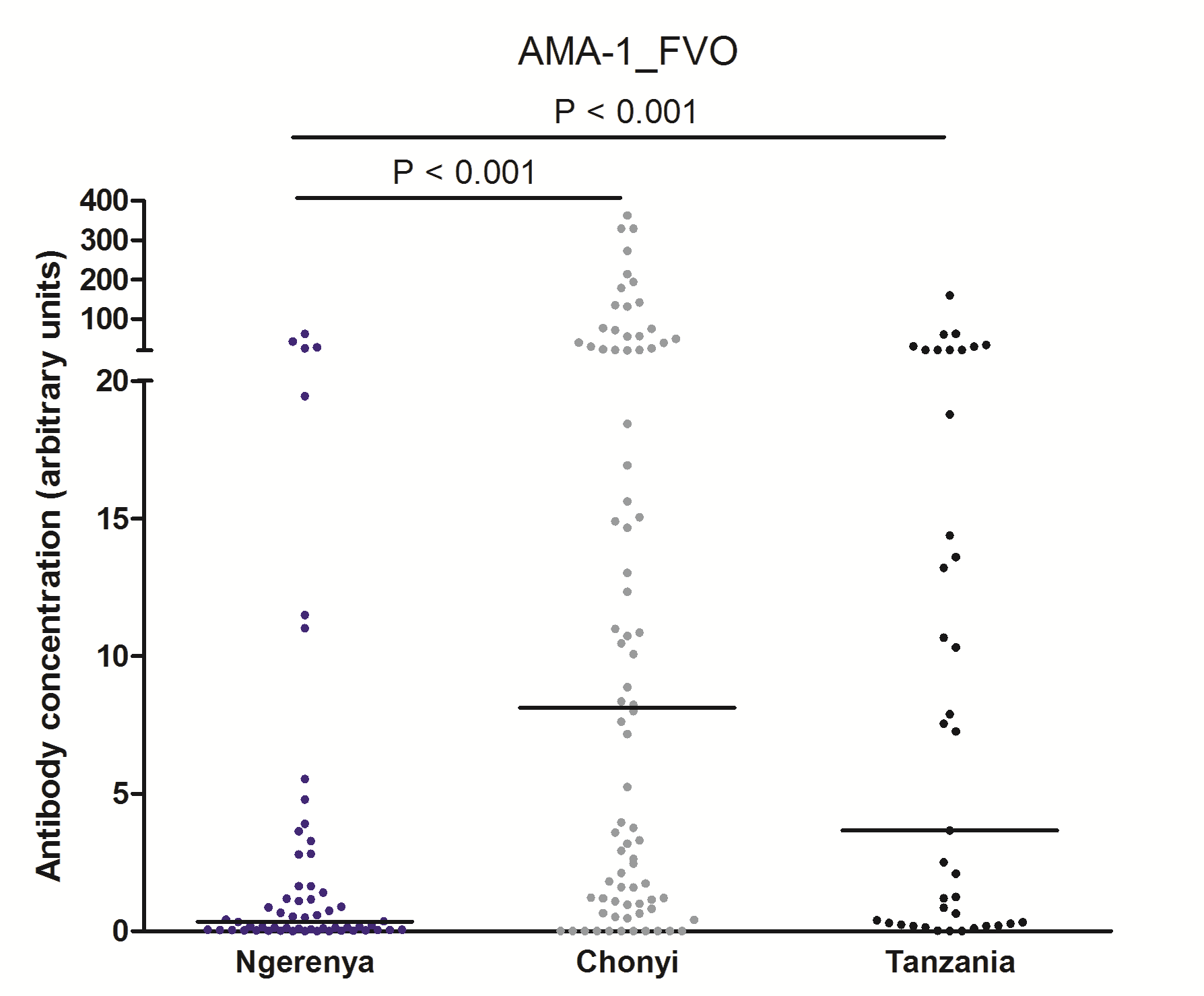** |
